# Supplementary material for: Learning diffractive optical communication around arbitrary opaque occlusions
Source: Nat Commun. 2023 Oct 26;14:6830. doi: 10.1038/s41467-023-42556-0 (PMC10603111; doi:10.1038/s41467-023-42556-0)
Supplement: Supplementary file 1 — Supplementary Information [file 41467_2023_42556_MOESM1_ESM.pdf]

Supplementary Information for

Learning Diffractive Optical Communication Around Arbitrary Opaque Occlusions

Md Sadman Sakib Rahman[1,2,3], Tianyi Gan[1,3], Emir Arda Deger[1], Çağatay Işıl[1,2,3], Mona Jarrahi[1,3], and Aydogan Ozcan[1,2,3]

[1]Electrical and Computer Engineering Department, University of California, Los Angeles, CA, 90095, USA
[2]Bioengineering Department, University of California, Los Angeles, CA, 90095, USA
[3]California NanoSystems Institute (CNSI), University of California, Los Angeles, CA, 90095, USA
[*]Corresponding author: ozcan@ucla.edu

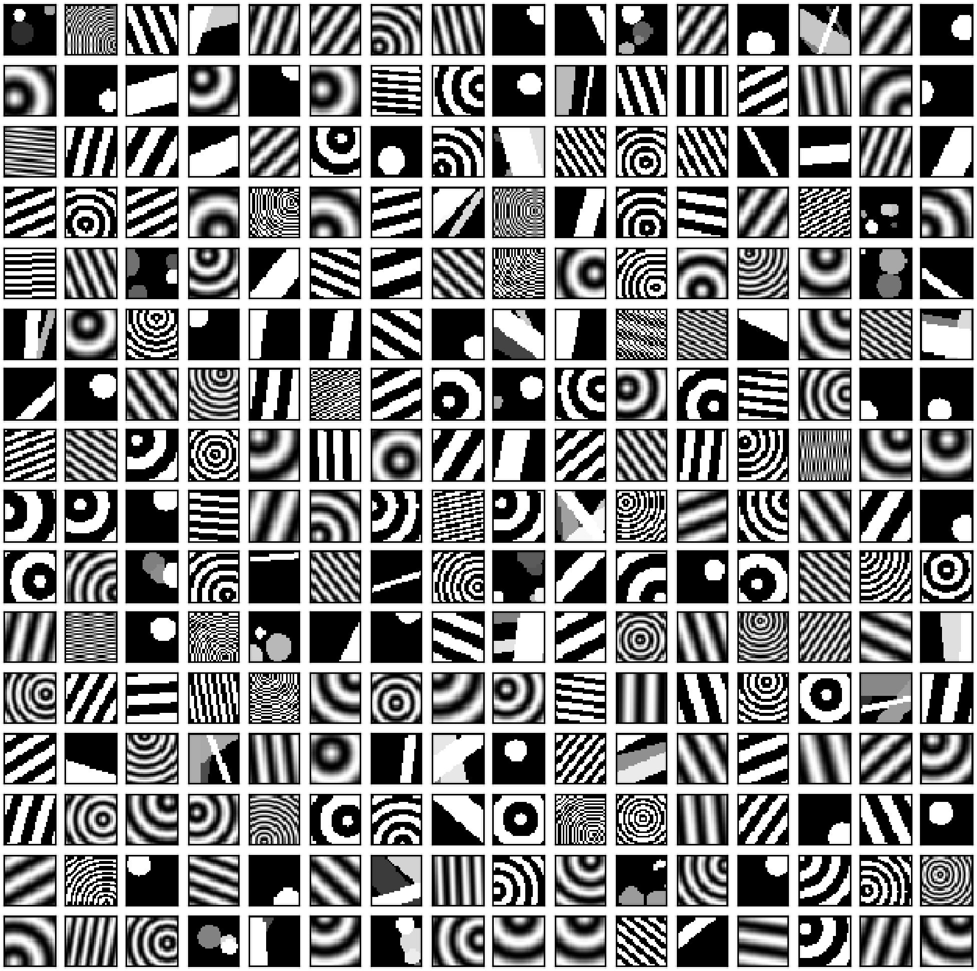

Fig. S1 Examples of the custom-prepared training images.

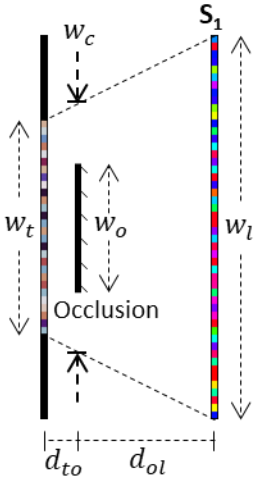

w_t: Transmitter aperture width
w_o: Occlusion width
w_l: Receiver aperture (diffractive layer-1) width
d_{to}: Distance from transmitter to occlusion
d_{ol}: Distance from occlusion to diffractive layer-1

w_c: Minimum occlusion width for which no light ray from the transmitter aperture reaches the diffractive decoder directly

$$w_c = w_t \frac{d_{ol}}{d_{to} + d_{ol}} + w_l \frac{d_{to}}{d_{to} + d_{ol}}$$

Fig. S2 The critical occlusion width w_c. For $w_o \geq w_c$, there is no direct path of light rays for communication between the transmitter and the receiver apertures.

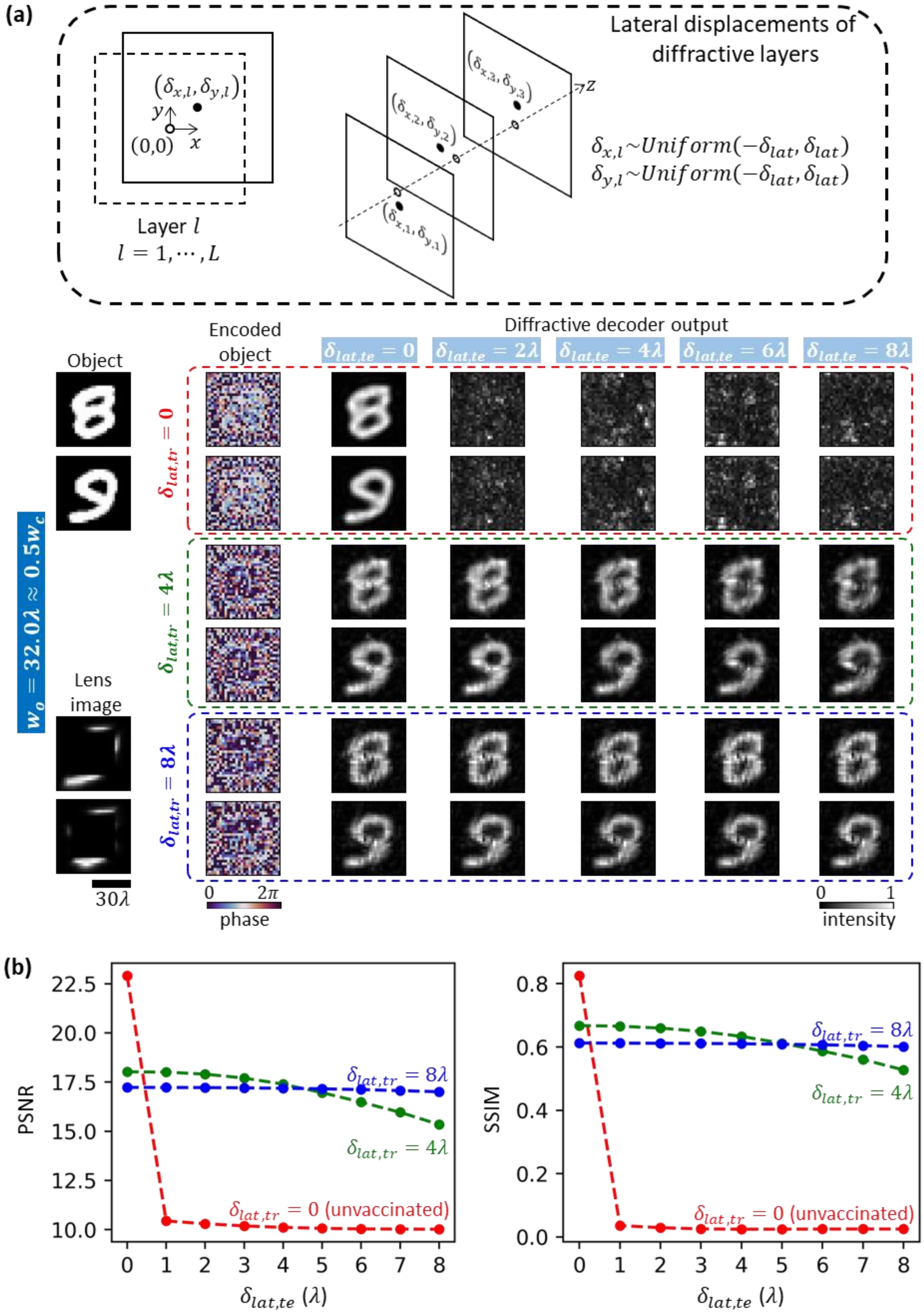

Fig. S3 Impact of lateral random misalignments of the layers of the diffractive decoder on the performance of our framework for communication around opaque occlusions. (a) Examples of the

diffractive decoder outputs at different levels of lateral random misalignments $\delta_{lat,te}$ during the testing for three designs trained with different levels of "vaccination" against such random misalignments ($\delta_{lat,tr} = 0$, $\delta_{lat,tr} = 4\lambda$, and $\delta_{lat,tr} = 8\lambda$). (b) Average PSNR and SSIM values of the diffractive decoder outputs as a function of $\delta_{lat,te}$ for the designs corresponding to $\delta_{lat,tr} = 0$, $\delta_{lat,tr} = 4\lambda$, and $\delta_{lat,tr} = 8\lambda$.

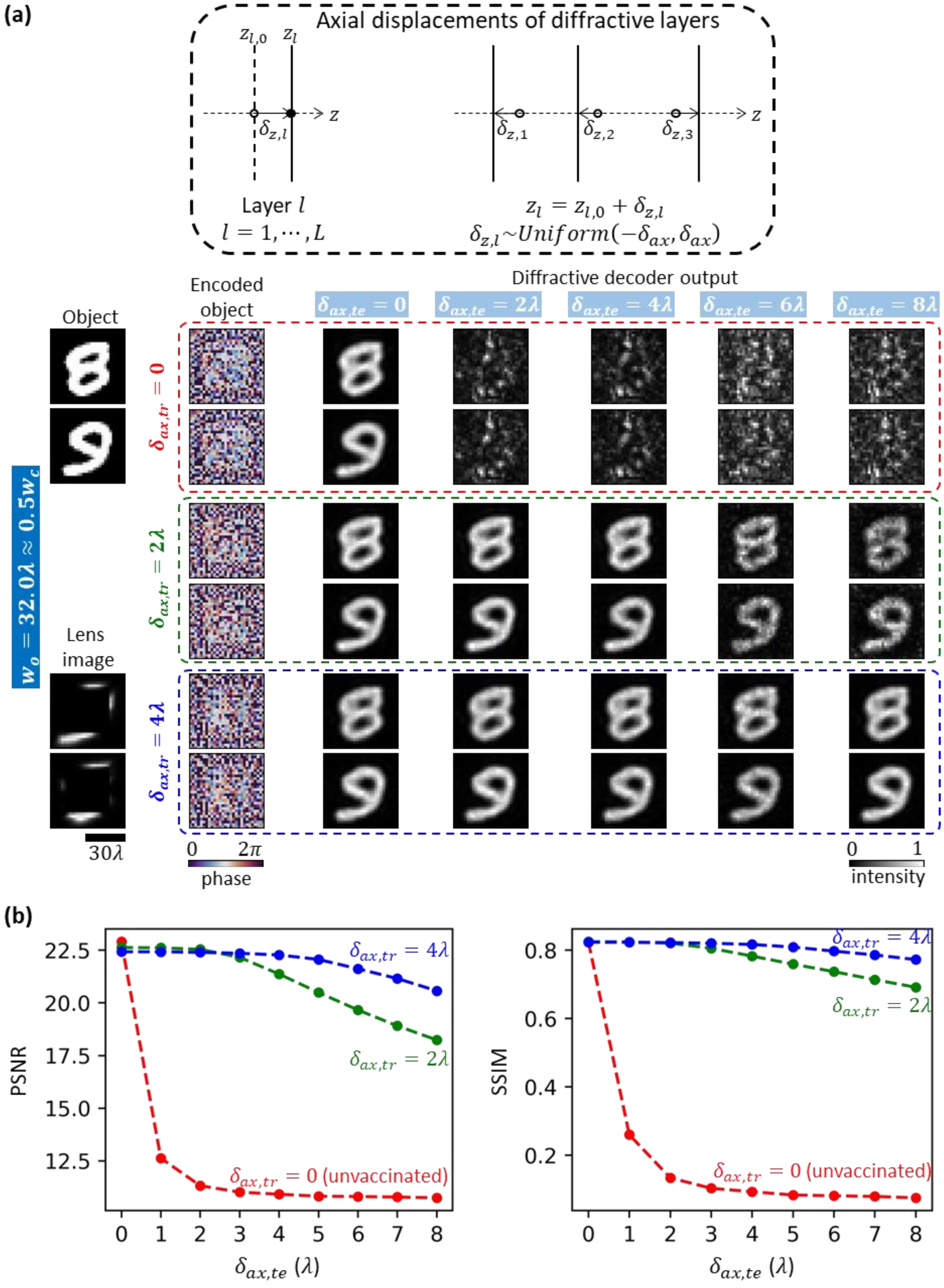

Fig. S4 Impact of axial random misalignments of the layers of the diffractive decoder on the performance of our framework for communication around opaque occlusions. (a) Examples of the

diffractive decoder outputs at different levels of axial random misalignments $\delta_{ax,te}$ during the testing for three designs trained with different levels of "vaccination" against such random misalignments ($\delta_{ax,tr} = 0$, $\delta_{ax,tr} = 2\lambda$, and $\delta_{ax,tr} = 4\lambda$). (b) Average PSNR and SSIM values of the diffractive decoder outputs as a function of $\delta_{ax,te}$ for the designs corresponding to $\delta_{ax,tr} = 0$, $\delta_{ax,tr} = 2\lambda$, and $\delta_{ax,tr} = 4\lambda$.

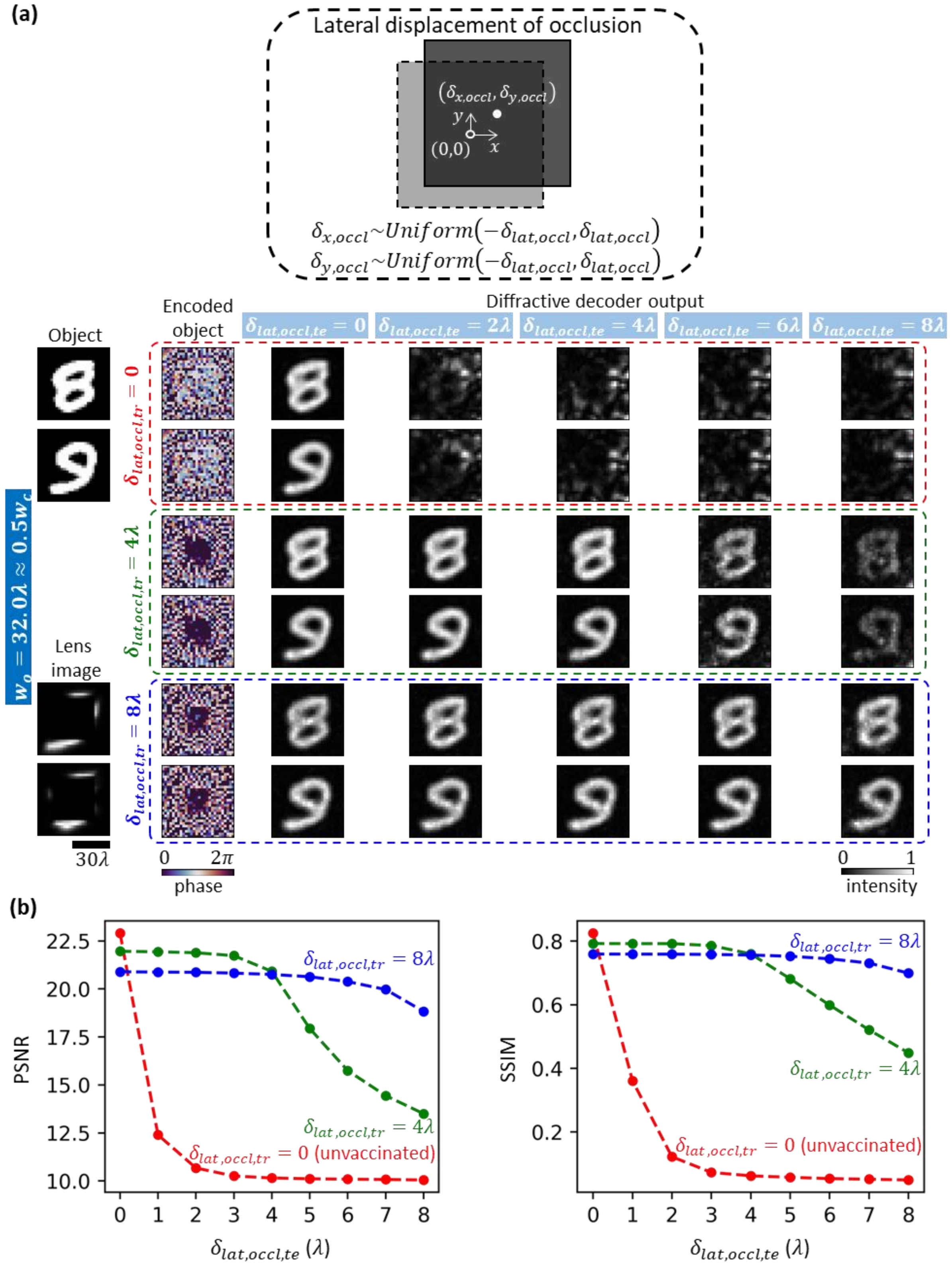

Fig. S5 Impact of lateral random displacements of the opaque occlusion on the performance of our framework for communication around opaque occlusions. (a) Examples of the diffractive decoder

outputs at different levels of lateral random displacements of the occlusion $\delta_{lat,occl,te}$ during the testing for three designs trained with different levels of "vaccination" against such random displacements of the occlusion ($\delta_{lat,occl,tr} = 0$, $\delta_{lat,occl,tr} = 4\lambda$, and $\delta_{lat,occl,tr} = 8\lambda$). (b) Average PSNR and SSIM values of the diffractive decoder outputs as a function of $\delta_{lat,occl,te}$ for the designs corresponding to $\delta_{lat,occl,tr} = 0$, $\delta_{lat,occl,tr} = 4\lambda$, and $\delta_{lat,occl,tr} = 8\lambda$.

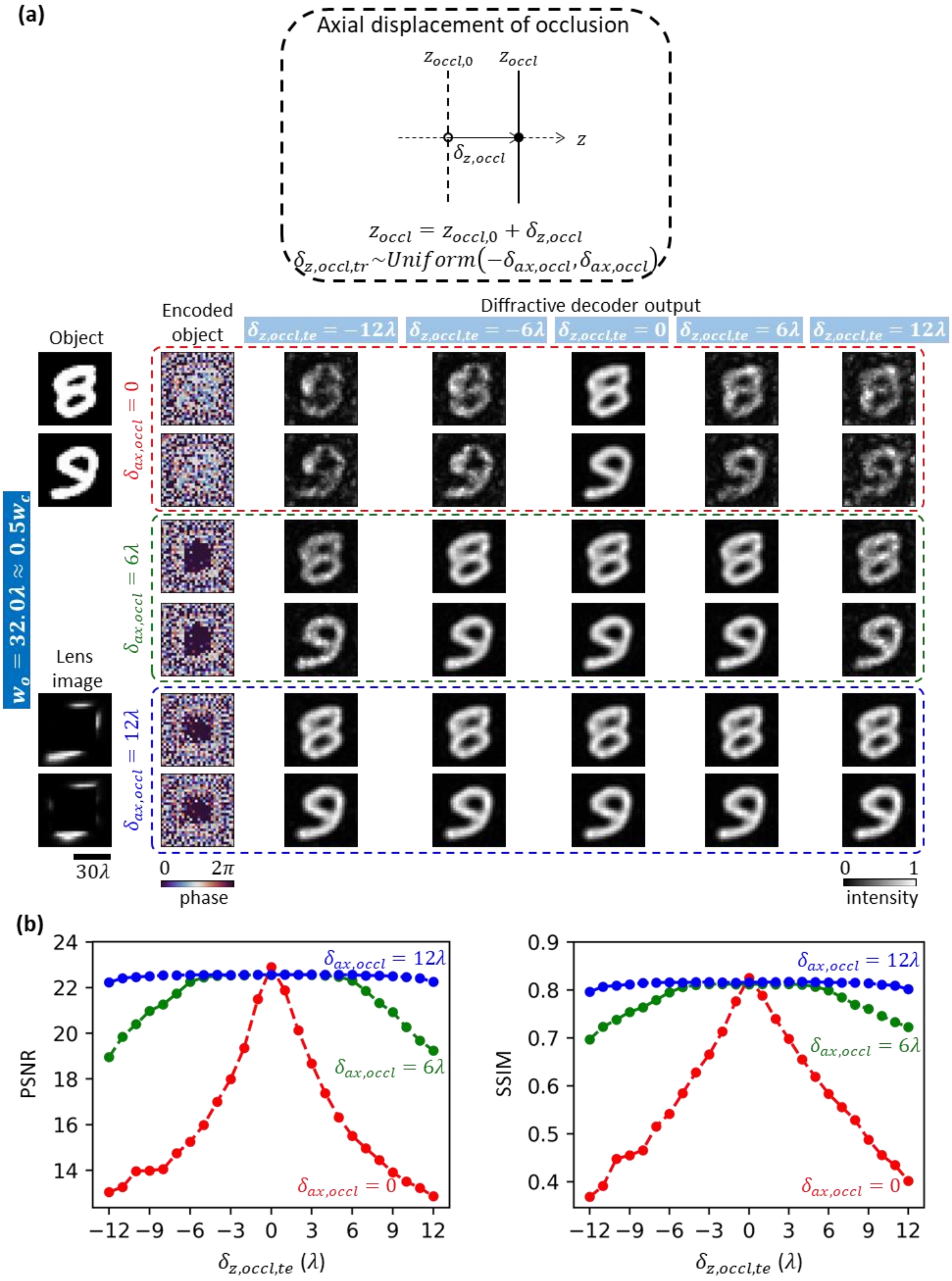

Fig. S6 Impact of axial random displacements of the opaque occlusion on the performance of our framework for communication around opaque occlusions. (a) Examples of the diffractive decoder

outputs at different axial displacements of the occlusion $\delta_{z,occl,te}$ during the testing for three designs trained with different levels of "vaccination" against random axial displacements of the occlusion ($\delta_{ax,occl} = 0$, $\delta_{ax,occl} = 6\lambda$, and $\delta_{ax,occl} = 12\lambda$). (b) Average PSNR and SSIM values of diffractive decoder outputs as a function of $\delta_{z,occl,te}$, for the designs corresponding to $\delta_{ax,occl} = 0$, $\delta_{ax,occl} = 6\lambda$, and $\delta_{ax,occl} = 12\lambda$.

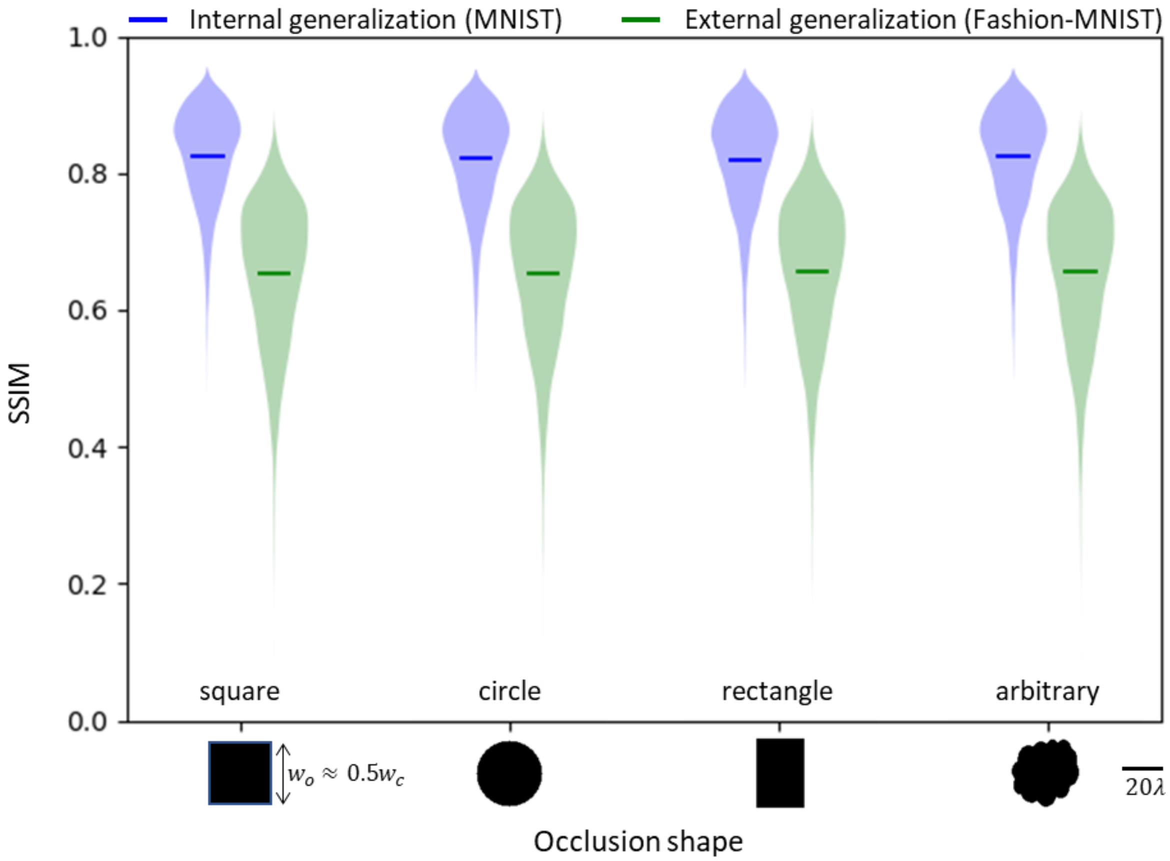

Fig. S7 Average SSIM values of the diffractive decoder outputs for the four designs of Fig. 8 (main text), calculated over 10,000 test images from the MNIST dataset (internal generalization) and 10,000 test images from the Fashion-MNIST dataset (external generalization).

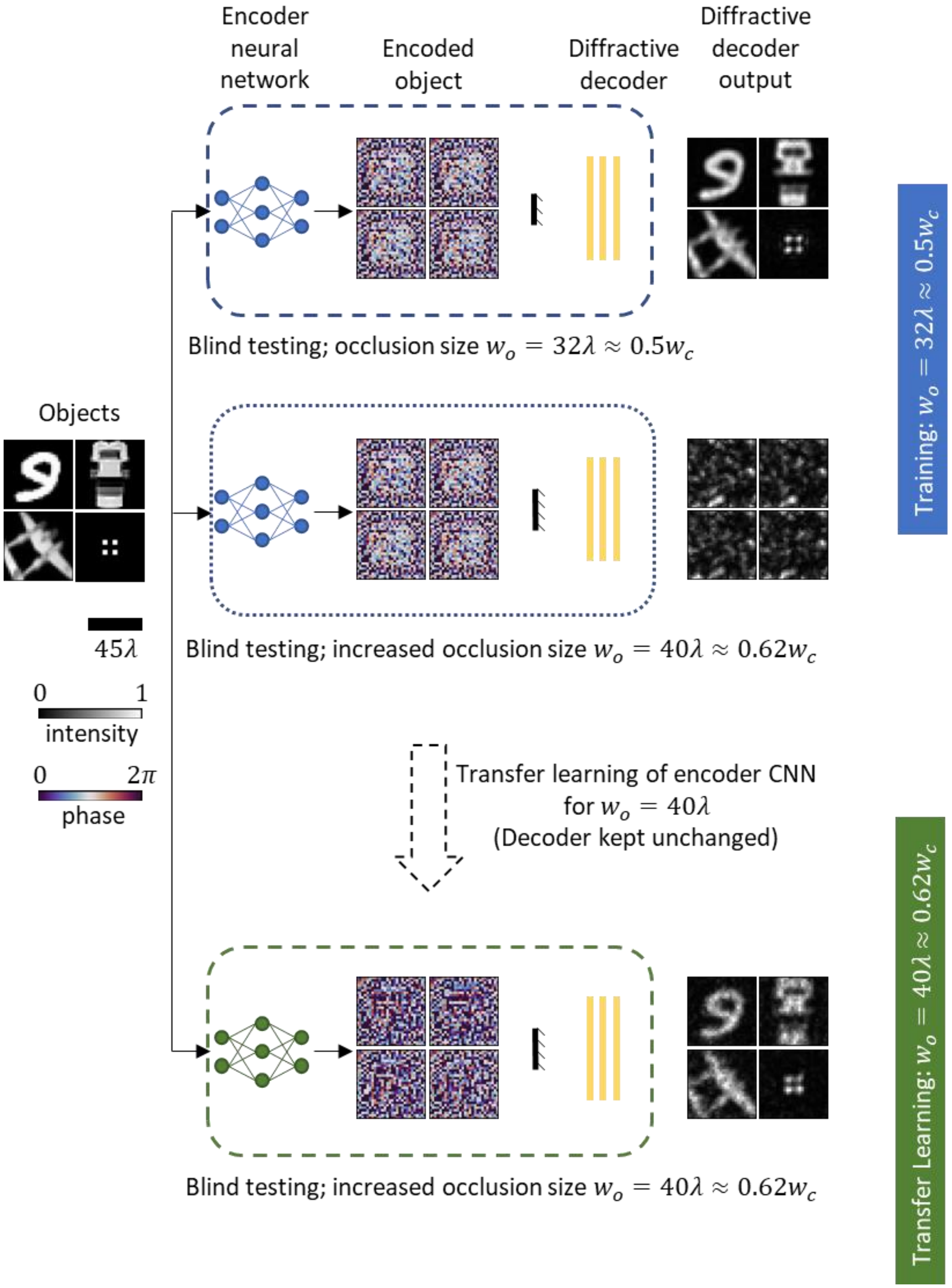

Fig. S8 Transfer learning of the CNN encoder at the transmitter, while the diffractive decoder at the receiver remains unchanged, for successful communication in case of an increase/change in the size of the opaque occlusion, obstructing the transmitter field-of-view.

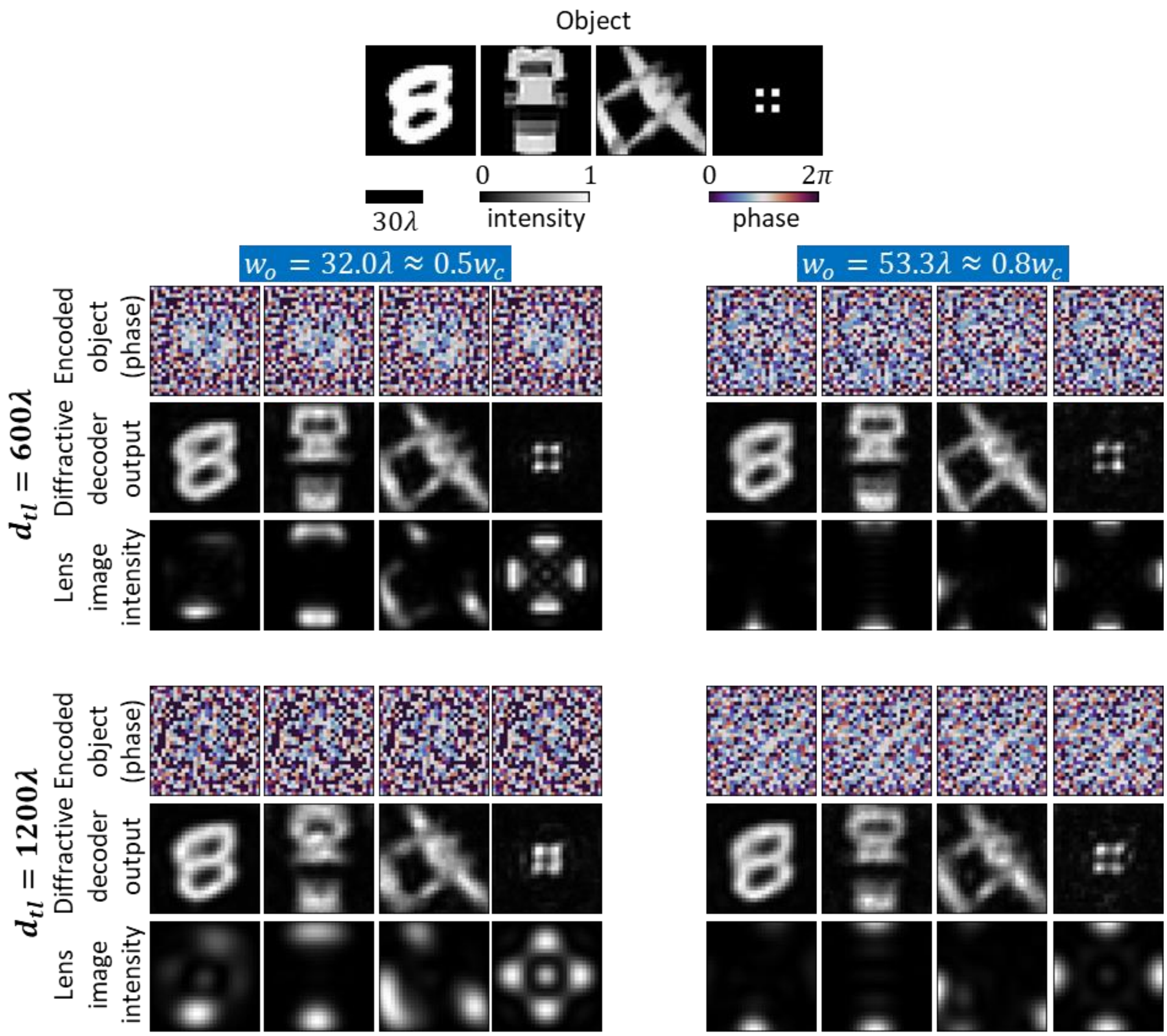

Fig. S9 Performance of the electronic encoder and diffractive decoder framework for communication around opaque occlusions for large values of the axial distance d_{tl} between the transmitter and the receiver apertures. The occlusion is assumed to be $d_{to} = d_{tl}/9$ distance away from the transmitter aperture.

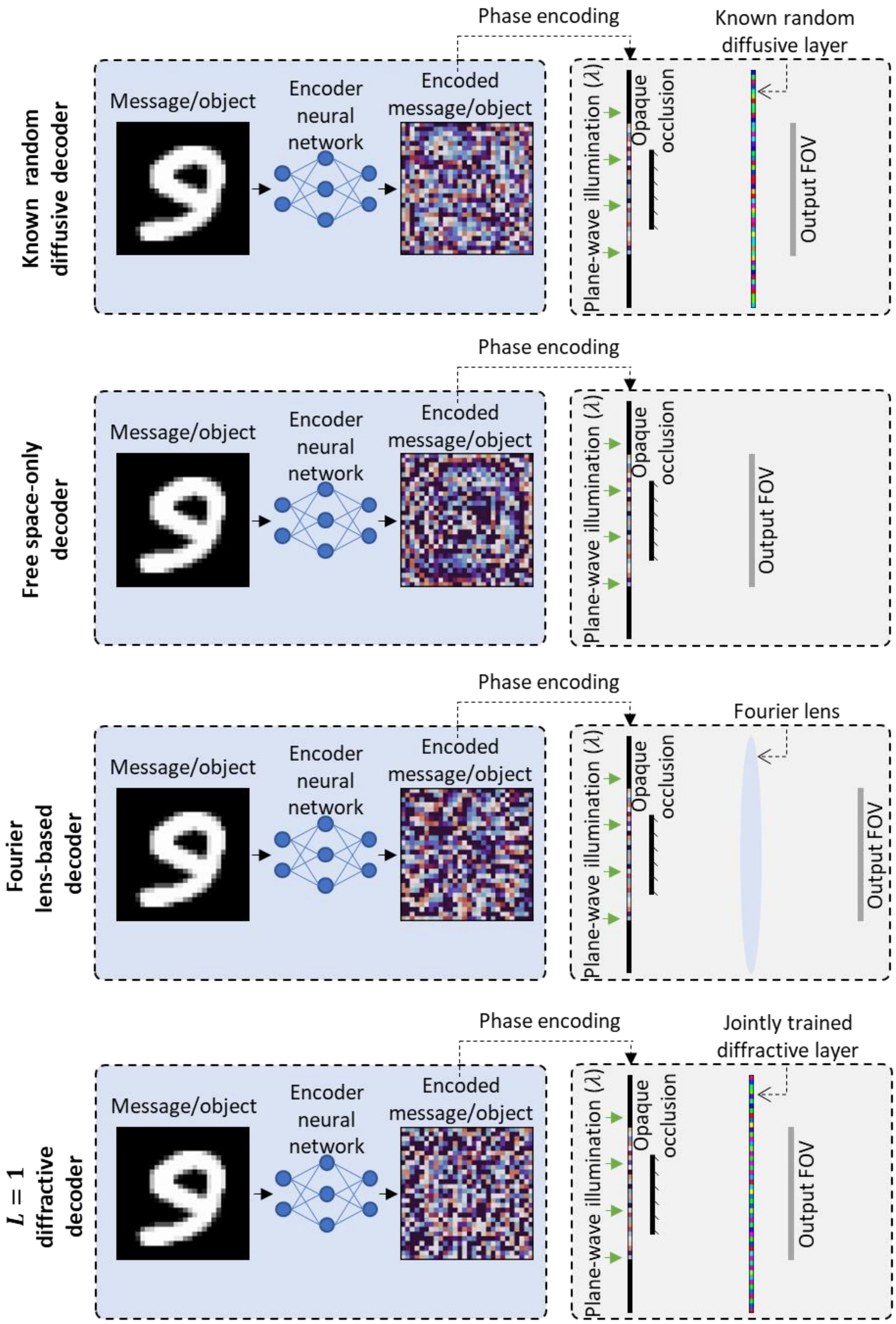

Fig. S10 Depiction of alternative schemes to diffractive all-optical decoding for communication around opaque occlusions. See Supplementary Fig. S11 for the comparative performance analysis of these different schemes for communication around opaque occlusions.

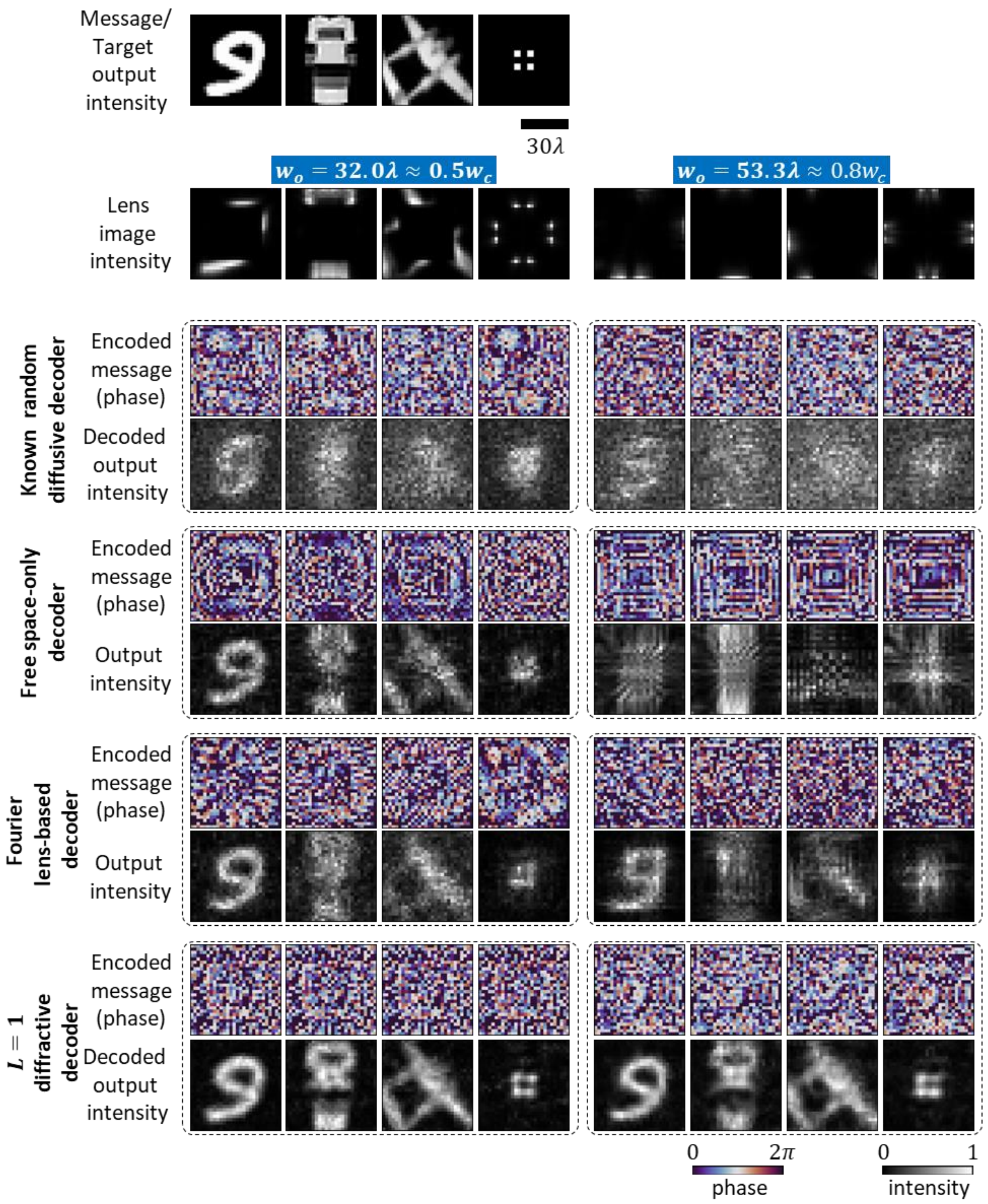

Fig. S11 The performances of a known random diffusive decoder, free space-only decoder, and Fourier lens-based decoder (see Supplementary Fig. S10) are compared against the performance of $L = 1$ layer diffractive decoder for two different sizes of opaque occlusion (w_o).
