# Supplementary material for: Learning diffractive optical communication around arbitrary opaque occlusions
Source: Nat Commun. 2023 Oct 26;14:6830. doi: 10.1038/s41467-023-42556-0 (PMC10603111; doi:10.1038/s41467-023-42556-0)
Supplement: Supplementary file 4 — Description of Additional Supplementary Files [file 41467_2023_42556_MOESM4_ESM.pdf]

Description of Additional Supplementary Files

Supplementary Video 1: Performance of an $L = 3$ design for communication around opaque occlusions as the shape of the opaque occlusion randomly changes within the occlusion plane, while the electronic encoder and the diffractive decoder remain the same. $r_{max} \approx 17.6\lambda$.

Supplementary Video 2: Performance of an $L = 3$ design for communication around opaque occlusions as the shape of the opaque occlusion randomly changes within the occlusion plane, while the electronic encoder and the diffractive decoder remain the same. $r_{max} \approx 29.3\lambda$.

Supplementary Video 3: Performance of an $L = 3$ design for communication around opaque occlusions as the shape of the opaque occlusion randomly changes within the occlusion plane, while the electronic encoder and the diffractive decoder remain the same. $r_{max} \approx 41.1\lambda$.
